# Psychosis in a primary hyperparathyroidism patient with mild hypercalcemia: A case report

**DOI:** 10.1097/MD.0000000000025248

**Published:** 2021-03-26

**Authors:** Koji Otsuki, Muneto Izuhara, Shoko Miura, Satoko Yamashita, Michiharu Nagahama, Maiko Hayashida, Sadayuki Hashioka, Tsuyoshi Miyaoka, Yukie Hotta, Yasuhiko Shimizu, Masatoshi Inagaki

**Affiliations:** a Department of Psychiatry, Faculty of Medicine, Shimane University, Izumo; b Matsue Aoba Hospital, Matsue; c Department of Otorhinolaryngology, Faculty of Medicine, Shimane University, Izumo, Japan.

**Keywords:** case report, mild hypercalcemia, parathyroidectomy, primary hyperparathyroidism, psychosis

## Abstract

**Introduction:**

Primary hyperparathyroidism (PHPT) is characterized by hypercalcemia and an elevated level of serum parathyroid hormone (PTH). PHPT presents with a complex set of renal, skeletal, and neuropsychological symptoms. Parathyroidectomy (PTX) is a radical treatment that is recommended for all physically symptomatic patients with PHPT. However, psychiatric symptoms are not considered as an indication for surgery. There remains an important issue from the view of perioperative management of whether PTX should be performed with the presence of uncontrolled psychiatric symptoms or deferred until severe psychiatric symptoms have been controlled. We report a case of mild hypercalcemia that caused severe psychosis in PHPT, which improved dramatically following PTX and resulted in successful postoperative management.

**Patient concern:**

Our patient was a 68-year-old Japanese woman. She was diagnosed with PHPT, which was triggered by mild hypercalcemia. She was due to receive an operation for osteoporosis and kidney stones. She had severe psychosis, despite medication. Blood examinations revealed mild hypercalcemia (10.4 mg/dL, 8.8–10.1 mg/dL) and elevated serum levels of intact PTH (184.0 pg/mL, 10–65 pg/mL).

**Diagnosis:**

She was diagnosed with severe psychosis caused by mild hypercalcemia in PHPT.

**Interventions:**

Although she was treated with 37.5 mg quetiapine and 2 mg risperidone daily, she was excessively sedated and rejected oral treatment. Therefore, we decided to perform the operation.

**Outcomes:**

Immediately following surgery, serum levels of calcium, and intact PTH were normalized. Her psychotic symptoms ceased completely 5 days after surgery.

**Conclusion:**

We emphasize that PHPT presents with various severe psychiatric symptoms, even in mild hypercalcemia. Psychiatric symptoms may be the only salient symptoms in PHPT, and thus clinicians should suspect PHPT in patients with psychiatric symptoms and mild hypercalcemia. Furthermore, PTX is recommended for PHPT—even in the presence of severe uncontrolled psychiatric symptoms, which carries risks for postoperative management—because psychiatric symptoms are expected to improve and good postoperative management is possible.

## 1 Introduction

Primary hyperparathyroidism (PHPT) is a common endocrine disease that is characterized by hypercalcemia and an elevated level of serum parathyroid hormone (PTH). The condition is caused by excessive secretion of PTH from one or more of the parathyroid glands.^[1]^ PHPT presents with a complex set of renal, skeletal, and neuropsychological symptoms. Elevated levels of PTH lead to persistent hypercalcemia, which often presents with subtle clinical symptoms.^[2]^

The psychiatric symptoms are rare in PHPT, the incidence has been reported approximately 4.2% among 405 PHPT patients.^[3]^ The psychiatric symptoms are divided into 3 patterns: confusional psychosis which was characterized by a change in consciousness ranging from drowsiness to stupor, paranoid psychosis with a clear sensorium which had severe depression and often paranoid delusions, pseudoneurotic form which showed a long history of complaints such as fatigue, lassitude, weakness, poor appetite, and constipation.^[3]^

Parks et al summarized the relationship between the degree of hypercalcemia and psychiatric symptoms.^[4]^ They reported that mild to moderate hypercalcemia (serum calcium level 10–14 mg/dL) is associated with depression, apathy, irritability, and lack of initiative and spontaneity. Severe hypercalcemia (serum calcium level >14 mg/dL) induces delirium with psychosis, catatonia, and lethargy.^[4]^

Parathyroidectomy (PTX) is a radical treatment and is recommended for all physically symptomatic patients with PHPT. PTX improves hypercalcemia and leads to an increase in bone mineral density and a reduction in nephrolithiasis. However, psychiatric symptoms are not included as an indication for surgery.^[2]^ There remains an important problem from the view of postoperative management of whether PTX should be performed with the presence of uncontrolled psychiatric symptoms or postponed until severe psychiatric symptoms have been controlled. Because of severe psychiatric symptoms, the inability to maintain postoperative rest makes safe postoperative management difficult. Therefore, psychosis associated with PHPT and psychosis co-existing with PHPT must be differentiated. We report a case of psychosis caused by mild hypercalcemia in PHPT, who dramatically improved with PTX.

## 2 Case report

Our patient was a 68-year-old Japanese woman. She was healthy by nature, but at the age of 60 years she was stricken with rheumatoid arthritis. Her husband died when she was 64 years’ old, but she was able to live on her own without any difficulties. At the age of 67, she had delirium when she was hospitalized for gastroenteritis, but she settled down after leaving the hospital. When she was 68 years’ old, her primary care doctor noted mild hypercalcemia and an increased level of intact parathyroid hormone (iPTH). She was examined and diagnosed with PHPT by an endocrinologist. She was scheduled for an operation for mild hypercalcemia, osteoporosis, and kidney stones. Her mental state was stable at the time of diagnosis. One week before visiting our hospital, she had insomnia, disorientation, auditory hallucinations, delusions, irritability, and disturbances of consciousness, so she visited our psychiatric department. Initially, we diagnosed delirium on the basis of these symptoms according to the Diagnostic and Statistical Manual of Mental Disorders, fifth edition,^[5]^ and suspected hypercalcemia as the cause. She was admitted to our hospital on the same day. Blood examinations revealed mild hypercalcemia (10.4 mg/dL, reference range, 8.8–10.1 mg/dL), elevated serum levels of iPTH (184.0 pg/mL, 10–65 pg/mL), and 1,25-(OH)_2_ vitamin D (69 pg/mL, 20–60 pg/mL). Thyroid function tests, chest x-ray, and brain magnetic resonance imaging (MRI) were all normal. She took 4 mg of prednisolone and 400 mg of etodolac daily.

We started treatment with antipsychotics (37.5 mg of quetiapine daily) for 1 week. The treatment improved her insomnia, disorientation, and consciousness disturbances; however, she continued to experience auditory hallucinations, delusions, and irritability. Therefore, we added 1 mg of risperidone daily. Two days later, she had sudden stupor, which continued for 2 days. After recovering from the stupor, she again experienced auditory hallucinations, delusions, and irritability. We suspected she had clear sensorium with psychosis, not delirium, because her disturbances of consciousness improved and her electroencephalography was normal. We increased doses of risperidone 1 mg to 2 mg daily, and she was excessively sedated. Therefore, she rejected oral treatment and severe psychotic symptoms continued for several days. During this time, we evaluated her psychiatric symptoms using the Brief Psychiatric Rating Scale (BPRS),^[6]^ which revealed high score of 57.

It was difficult to continue medication because she was excessively sedated due to increased doses of antipsychotics and refused to take the pills. Postoperative management would be difficult if her psychotic symptoms continued following surgery. In such cases, the operation should be postponed until psychotic symptoms are ameliorated. However, we also considered that her psychotic symptoms may have been caused by PHPT and thus, surgical treatment would alleviate these symptoms. Therefore, we performed the operation as planned.

Immediately after surgery, the serum levels of calcium and iPTH were normalized. Histologic diagnosis of the surgical sample was parathyroid adenoma. Her psychotic symptoms ceased completely, 5 days after surgery. Her BPRS score decreased from 57 to 18. Table 1 summarizes the changes in biochemical parameters and psychiatric evaluation after PTX. She has had no psychiatric complaints for over 1 year after surgery.

**Table 1:** The changes in biochemical parameters and psychiatric evaluation after parathyroidectomy.

	Before	After
Serum calcium (8.8–10.1 mg/dL)	10.4	9.0
Intact PTH (10–65 pg/mL)	184.0	24.3
1,25-(OH)_2_ vitamin D (20–60 pg/mL)	69	47
BPRS	57	18

BPRS = brief psychiatric rating scale, PTH = parathyroid hormone.

## 3 Discussion

We report a case of mild hypercalcemia that caused severe psychosis in PHPT, which improved dramatically with PTX. After surgery, there was an immediate normalization of calcium and iPTH serum levels. The psychotic symptoms subsided 5 days after surgery. Taken together, we concluded that the psychotic symptoms were caused by mild hypercalcemia with PHPT. PTX was an effective treatment for psychotic symptoms caused by mild hypercalcemia with PHPT. We had concerns regarding whether to operate because of the severe psychotic symptoms; however, the decision to perform the PTX resulted in a good outcome.

Interestingly, in our case, severe psychotic symptoms were induced by only a slight increase in hypercalcemia. In general, the severity of psychiatric symptoms is related to the degree of hypercalcemia. However, in PHPT especially, the correlation between the degree of hypercalcemia and severity of psychiatric symptoms is inconclusive.^[4,7]^ McDonald et al^[8]^ reported that even mild hypercalcemia can be harmful if there are vulnerabilities, including old age, a history of hypertension, white matter lesions on computed tomography scans, recent bereavements, and possibly a degree of persistent cognitive impairment. Our case had mild hypercalcemia but was not aged >70 years and did not have hypertension, abnormal findings in brain MRI, or cognitive impairment. The mechanisms underlying psychiatric symptoms in PHPT are still unclear and warrant further investigation.

Importantly, in our patient, PTX was effective in treating severe psychotic symptoms in a patient with mild hypercalcemia. Although PTX is recommended for all physically symptomatic patients with PHPT, adapting treatment on the basis of psychiatric symptoms is not common practice. In regard to the relationship between PTX and psychiatric symptoms, several randomized controlled trials (RCT) have reported varied results.^[9–12]^ To our knowledge, four RCTs have investigated the effects of PTX on mental status in PHPT using the short form-36 general health survey (SF-36).^[9–12]^ Three RCTs showed that PTX improved some of the items of the SF-36^[9,11,12]^, whereas one RCT showed no benefit.^[10]^ A meta-analysis concluded that PTX has no substantial effects on neuropsychiatric symptoms.^[13]^ Meanwhile, several case reports showed recovery of acute psychotic symptoms in patients with PHPT following PTX.^[7,14,15]^ Further research is needed to determine whether psychiatric symptoms are to be included as indications for PTX surgery. Based on our experience, we recommend PTX for patients with PHPT who experience severe psychiatric symptoms, since these symptoms are expected to improve and successful postoperative management is possible.

In this case, antipsychotics failed to control psychiatric symptoms but were improved by PTX. The cause of the psychiatric symptoms was determined to be mild hypercalcemia in PHPT; however, it was difficult to establish whether the symptoms were associated with or were independent of PHPT when antipsychotics were shown to be ineffective. Therefore, it is advisable to differentiate psychiatric symptoms that are independent of PHPT from those that are associated with PHPT by using serum calcium levels, and PTX should be performed if appropriate.

## 4 Conclusions

In conclusion, we report a case of mild hypercalcemia that caused severe psychotic symptoms in PHPT, which improved dramatically following PTX. We highlight the following two points. First, various severe psychiatric symptoms can be present in PHPT patients, even in mild hypercalcemia. Therefore, the clinicians should assess for PHPT in patients with psychiatric symptoms and mild hypercalcemia. Second, PTX might be recommended for PHPT even in the presence of severe uncontrolled psychiatric symptoms, because psychiatric symptoms might be expected to improve and good postoperative management is possible.

## Acknowledgments

We thank Sarina Iwabuchi, PhD, from Edanz Group (https://en-author-services.edanzgroup.com/ac) for editing a draft of this manuscript.

## Author contributions

KO described the case. KO wrote the draft and performed the literature search. MI, SM, SY, MN, MH, SH, TM, YH, YS and MI contributed to the conception or design of the work. All authors read and approved the final manuscript.

**Conceptualization:** Koji Otsuki.

**Supervision:** Maiko Hayashida, Sadayuki Hashioka, Tsuyoshi Miyaoka, Yukie Hotta, Yasuhiko Shimizu, Masatoshi Inagaki.

**Validation:** Muneto Izuhara, Shoko Miura, Satoko Yamashita, Michiharu Nagahama.

**Writing – original draft:** Koji Otsuki.

**Writing – review & editing:** Koji Otsuki, Masatoshi Inagaki.
